# Prevalence of actionable pharmacogenomic variants in Brazilian patients with cancer

**DOI:** 10.3389/fphar.2026.1736887

**Published:** 2026-03-02

**Authors:** Jaqueline B. Schuch, Mariana R. Botton, Angélica C. De Baumont, Giovana Curzel, Nathan A. Cadore, Cláudia Bordignon, Mahira L. Rosa, Vitor F. Vasconcellos, Lilian A. R. Barros, Cristiano P. Souza, Williams F. Barra, Daniela L. C. Louzeiro, Alessandra Notari, Juliana J. de Menezes, Pedro E. R. Liedke, Gláucio A. Bertollo, Aline B. L. Gongora, Henrique G. Ascenco, Eduardo Kowalski-Neto, Christina P. Oppermann, Gustavo Werutsky, Edilmar M. Santos, Flavio S. Brandão, Ruffo Freitas-Junior, Angélica Nogueira-Rodrigues, André L. C. Mancini, Marina Bessel, Gabriel S. Macedo, Daniela D. Rosa

**Affiliations:** 1 Hospital Moinhos de Vento, Porto Alegre, Brazil; 2 Hospital de Clínicas de Porto Alegre, Porto Alegre, Brazil; 3 Universidade Federal do Rio Grande do Sul, Programa de Pós-Graduação em Genética e Biologia Molecular, Porto Alegre, Brazil; 4 Hospital Universitário Cassiano Antônio Moraes, Vitória, Brazil; 5 Instituto Brasileiro de Controle do Câncer, São Paulo, Brazil; 6 Hospital de Câncer de Barretos, Barretos, Brazil; 7 Núcleo de Pesquisas em Oncologia, Universidade Federal do Pará, Belém, Brazil; 8 Hospital de Oncologia Dr. Tarquinio Lopes Filho, São Luís, Brazil; 9 Hospital Escola da Universidade Federal de Pelotas/Empresa Brasileira de Serviços Hospitalares (EBSERH), Pelotas, Brazil; 10 Hospital Nossa Senhora da Conceição, Porto Alegre, Brazil; 11 Associação Feminina de Educação e Combate ao Câncer (AFECC), Hospital Santa Rita de Cássia, Vitória, Brazil; 12 Hospital do Câncer UOPECCAN, Cascavel, Brazil; 13 Hospital Universitário Maria Aparecida Pedrossian, Campo Grande, Brazil; 14 Hospital Calixto Midlej Filho, Santa Casa de Misericórdia de Itabuna, Itabuna, Brazil; 15 Faculdade de Medicina, Universidade Estadual de Santa Cruz, Ilhéu, Brazil; 16 Hospital Fêmina, Porto Alegre, Brazil; 17 Hospital São Lucas PUCRS, Porto Alegre, Brazil; 18 Liga Norte Riograndense Contra o Câncer, Natal, Brazil; 19 Santa Casa de Belo Horizonte, Belo Horizonte, Brazil; 20 Hospital do Câncer Araújo Jorge da Associação de Combate ao Câncer em Goiás, Goiânia, Brazil; 21 Universidade Federal de Minas Gerais, Belo Horizonte, Brazil; 22 Hospital Universitário Getúlio Vargas, Manaus, Brazil; 23 Instituto D'Or de Pesquisa e Ensino (IDOR), São Paulo, Brazil; 24 Faculdade de Medicina, Universidade Federal do Rio Grande do Sul, Porto Alegre, Brazil

**Keywords:** breast neoplasms, exome sequencing, pharmacogenetics, pharmacogenomics, prostate neoplasms

## Abstract

**Introduction:**

Pharmacogenomic (PGx) variants can influence drug efficacy and safety, yet their prevalence in Latin American populations with cancer is underexplored. Our aim is to characterize the frequency and phenotypic distribution of actionable pharmacogenes in Brazilian patients with metastatic prostate cancer (MPC) and Human Epidermal Growth Factor Receptor 2 (HER2)-positive breast cancer (BC).

**Methods:**

This analysis included 452 patients (259 BC, 193 MPC) from a multicenter study across 19 Brazilian sites. Exome sequencing was performed, and PGx variants were analyzed using the Pharmacogenomics Clinical Annotation Tool (PharmCAT) following the Clinical Pharmacogenetics Implementation Consortium (CPIC®) guidelines. Genotypes, star alleles, and predicted phenotypes were reported for 15 clinically relevant pharmacogenes.

**Results:**

Actionable PGx phenotypes were detected in 99.33% of participants. The decreased-function *ABCG2* rs2231142 T allele occurred at 8.96%, and the *VKORC1* rs9923231 T allele at 32.63%. In *SLCO1B1*, normal function predominated (63.11%), with 21.11% exhibiting decreased function. Normal metabolizer phenotypes were most frequent in *CYP2C19* (45.35%), *CYP2C9* (70.51%), and *CYP3A4* (94.62%), whereas *CYP2B6* was dominated by intermediate metabolizers (43.02%) and *CYP3A5* by poor/intermediate metabolizers (93.79%). Normal diplotypes predominated in thiopurine-related genes (*NUDT15*: 92.92%; *TPMT*: 88.72%), although nonfunctional alleles were observed. In *UGT1A1*, decreased-function alleles accounted for approximately 37% of participants. Clinically relevant *DPYD* and *RYR1* variants were rare (<2.0%).

**Conclusion:**

Nearly all Brazilian patients with cancer carried at least one actionable PGx variant, highlighting the potential impact of PGx-guided therapy in oncology. These results underscore the value of integrating pharmacogenomic strategies into clinical practice in Brazil.

## 1 Introduction

Pharmacogenomics (PGx) involves the understanding of individual responses to drug therapies, including efficacy and toxicity, by analyzing the effects of genetic variability on the metabolism of drugs and prodrugs (Relling and Evans, 2015). This individual variability is of major clinical importance due to its potential association with drug efficacy and adverse drug reactions, ultimately affecting patient care and causing substantial health issues. Large-scale genotyping studies have shown that nearly all individuals carry at least one PGx variant that could impact outcomes related to medication use (Bush et al., 2016; Ji et al., 2016; Lanillos et al., 2022; McInnes et al., 2021; Van Driest et al., 2014).

Drug-response phenotypes can be partly attributed to individual genetic variability. A well-established example involves dihydropyrimidine dehydrogenase (*DPYD*) variants, which affect the metabolism of fluoropyrimidines in patients with colorectal cancer. Pathogenic variants in this gene are strongly associated with an increased risk of potentially life-threatening toxicity during treatment with agents such as 5-fluorouracil or capecitabine (Amstutz et al., 2018; NCCN, 2025). For this reason, international guidelines, including those from the National Comprehensive Cancer Network (NCCN), recommend *DPYD* testing in specific clinical settings. Other clinically relevant PGx applications include the assessment of uridine diphosphate glucuronosyltransferase 1A1 (*UGT1A1*) polymorphisms, associated with irinotecan-induced toxicity, and thiopurine S-methyltransferase (*TPMT*) and nudix hydrolase 15 (*NUDT15*) variants, predictive of adverse reactions to thiopurines used in certain hematologic malignancies (Gammal et al., 2016; Relling et al., 2019). These advances illustrate how the integration of PGx into cancer care can optimize therapeutic efficacy while improving patient safety (NCCN, 2025; Varughese et al., 2020).

Several challenges hinder the implementation of PGx, among which extraction and analysis of genomic variants are major obstacles (Klein and Ritchie, 2018). For instance, the cytochrome P450 (*CYP*) gene family is the most important gene family in PGx, as *CYP* genes are highly polymorphic. These genes encode cytochrome P450 proteins and are relevant in 10%–20% of all drug therapies (Zhou and Lauschke, 2022). To address the challenges in PGx assessment, specific PGx tools have been developed to identify PGx genotypes, infer haplotypes, and predict phenotypes. One such tool is the Pharmacogenomics Clinical Annotation Tool (PharmCAT), which adheres to the most recent PGx guidelines and uses a predictive framework to determine haplotypes in 18 actionable pharmacogenes, as well as the associated phenotypes (Klein and Ritchie, 2018).

Population admixture adds further complexity to the implementation of PGx. In admixed populations, the absence of comparable groups with relatively homogeneous genetic ancestry complicates the interpretation of results and hinders the implementation of genomic medicine into clinical practice, requiring specific genomic studies of these individuals. The genetic composition of the Brazilian population, with ancestry contributions from African, Native American, and European populations, has likely contributed to a genetically admixed population that remains underrepresented in public genomic reference databases (Mychaleckyj et al., 2017; Rodrigues De Moura et al., 2015; Secolin et al., 2019). A recent large-scale genomic study of the Brazilian population identified more than 8 million previously unreported genetic variants, highlighting both its extensive genetic diversity and the significant underrepresentation of admixed populations in global genomic databases (Nunes et al., 2025). Another study of individuals from the Southeast of Brazil investigated 38 pharmacogenes and found that 98% of participants carried at least one high-risk genotype-predicted phenotype, although a search for novel alleles was not included (Bertholim-Nasciben et al., 2023).

In the present study, we investigated the genetic frequency and phenotypic variability of actionable pharmacogenes in the Brazilian population. We used exome sequencing to identify variants in protein-coding pharmacogenes, enabling the large-scale study of variants, in men with metastatic prostate cancer (MPC) and women with Human Epidermal Growth Factor Receptor 2 (HER2)-positive breast cancer (BC). PGx variant selection was analyzed and interpreted according to the Clinical Pharmacogenetics Implementation Consortium (CPIC®) guidelines and implemented using PharmCAT, based on evidence from the Clinical Pharmacogenomic (ClinPGx) database and genomic data.

## 2 Materials and methods

### 2.1 Study design and participants

This is a secondary analysis of data derived from Onco-Genomas Brasil, a multicenter study conducted in 19 health facilities distributed among the 5 macro-regions of Brazil, ensuring broad geographic representation across the national territory. The sample size was calculated to estimate the prevalence of germline genetic alterations in cancer predisposition genes and was not powered for the detection of rare variants. Therefore, rare variants identified in this study are reported descriptively and should be interpreted as exploratory findings. The Onco-Genomas Brasil study is part of the Brazilian Genome Program (*Programa Genomas Brasil*) of the Ministry of Health (*Ministério da Saúde*). All participants received care in public hospitals through the Brazilian Unified Health System (*Sistema Único de Saúde - SUS*).

The sample consisted of 259 women with locally advanced HER2-positive BC and 193 men with MPC. The inclusion criteria for women with BC were age ≥18 years, histologically confirmed breast carcinoma with HER-2 overexpression (a score of 3+ or 2+ on immunohistochemistry with positive *in situ* hybridization), clinical stage II or III according to the American Joint Committee on Cancer (AJCC) classification, and use of neoadjuvant treatment with chemotherapy plus trastuzumab. For men with MPC, the inclusion criteria were age ≥18 years, histologically confirmed prostate adenocarcinoma, and clinical stage IV according to the AJCC classification.

Demographic data were collected from participant interviews and/or medical records. Ethno-racial self-identification was obtained through interviews and classified according to the categories defined by the Brazilian Institute of Geography and Statistics (IBGE): (1) White; (2) Black; (3) Mixed; (4) Asian; and (5) Indigenous. The detailed study protocol has been published previously (Schuch et al., 2024). Descriptive data for continuous variables are expressed as mean and standard deviation (SD) or median and interquartile range (IQR), depending on the data distribution. Categorical data are expressed as absolute and relative frequencies.

The study protocol was registered at ClinicalTrials.gov (NCT05306600) and approved by the research ethics committee of the coordinating center, Hospital Moinhos de Vento (CAAE 55457122.3.1001.5330), and by the local ethics committees of all participating centers. Written informed consent was obtained from all participants before their inclusion in the study.

### 2.2 Sample processing

Peripheral blood samples were collected from each participant and transported to the coordinating center for processing. Germline deoxyribonucleic acid (DNA) was extracted using the QIAamp DNA Blood Kit (Qiagen). The minimum sample quality parameters were defined as an absorbance ratio of 260:280 between 1.8 and 2.0 (NanoDrop) and a minimum total yield of 250 nanogram (ng), quantified with the Qubit™ 1X dsDNA High Sensitivity Kit (Invitrogen™, ref Q33231) using the Qubit™ 4 Fluorimeter (Invitrogen™, ref Q33238). These criteria were adopted to optimize the performance of exome sequencing.

Next-generation sequencing (NGS) was performed using the Illumina NovaSeq 6,000 platform. Library preparation was conducted with the Twist Library Preparation EF 2.0 Kit, target enrichment with the Twist Target Enrichment Standard Hybridization v2 Kit, and sequencing with the NovaSeq 6000 S4 Reagent Kit. The quality parameters for NGS included an average size of the DNA library obtained by Tapestation between 375 and 450 bp, and a minimum size of the sequenced target DNA fragment between 100 and 150 bp. Each sample achieved at least 50X mean coverage, a minimum Q30 of 85%, and contamination levels below 2%. Across all samples included in the analysis, the proportion of bases covered at >20X was 98.7% ± 0.08%, and the proportion of bases with Q30 or higher was 91.2% ± 1.4%. Sequence alignment and mapping were performed using Dynamic Read Analysis for GENomics (DRAGEN) v.3.10.4, using the Genome Reference Consortium Human Build 38 (GRCh38). Variant calling and quality filtering were performed using the DRAGEN pipeline with hard-filtering. The DRAGEN Joint Genotyping pipeline was used to merge and perform joint genotype calling of each sample into a multi-sample variant call format (VCF) file containing data from all germline samples and approximately 488,000 variants. Only variants marked as PASS after DRAGEN’s standard hard-filtering and joint genotyping were retained for pharmacogenomic analyses. The inference of the haplotypic phase from genomic data was obtained with Segmented HAPlotype Estimation and Imputation Tools version 5 (ShapeIT5) using the phase_common option, employing a cohort-based phasing framework without the use of an external reference panel, and the genetics maps in b38 for whole-genome sequencing chunks supplied by the tool (Hofmeister et al., 2023).

### 2.3 Pharmacogenomic analyses

The PharmCAT software was used to extract relevant PGx variants, infer diplotypes/genotypes, and predict phenotypes according to the CPIC® recommendations (Klein and Ritchie, 2018). The GRCh38 assembly was used as a reference. For each germline sample, a report was generated comprising genotype, allele functionality, and phenotype for the following genes: ATP Binding Cassette Subfamily G Member 2 (*ABCG2*), Interferon Lambda 3/4 (*IFNL3/4*), Vitamin K Epoxide Reductase Complex Subunit 1 (*VKORC1*), Solute Carrier Organic Anion Transporter Family Member 1B1 (*SLCO1B1*), Cytochrome P450 family genes (*CYP2B6*, *CYP2C19*, *CYP2C9*, *CYP3A4*, *CYP3A5*, *CYP4F2*), *NUDT15*, *TPMT*, *UGT1A1*, *DPYD*, and Ryanodine Receptor 1 (*RYR1*). Cytochrome P450 Family 2 Subfamily D Member 6 (*CYP2D6*) was not assessed due to the complexity of accurately detecting copy number variations in this gene. Consistent with PharmCAT recommendations, *CYP2D6* genotyping using WES is not supported in the standard pipeline because of the presence of highly homologous pseudogenes and frequent structural rearrangements, requiring specialized analytical approaches or long-read sequencing for reliable characterization (Sangkuhl et al., 2020). If multiple genotype combinations were generated for the positions of interest, additional analyses were performed. The 15 pharmacogenes included in this analysis were selected based on their clinical relevance and actionability, guided by recommendations from established pharmacogenomic guidelines (e.g., CPIC and Dutch Pharmacogenetics Working Group - DPWG) and evidence curated in pharmacogenomic databases such as ClinPGx. These genes harbor well-characterized functional variants with strong levels of evidence and validated genotype–phenotype relationships, supporting their potential clinical implementation across multiple therapeutic areas. A complete list of variants analyzed using this approach is provided in Supplementary Table S1. VCF files were analyzed to identify previously unreported alleles not yet included in the Pharmacogene Variation (PharmVar) database (Gaedigk et al., 2018). Key pharmacogenetic non-coding variants, including regulatory and promoter markers, were adequately covered with high genotype quality through off-target capture; their respective average coverage and call rates are provided in Supplementary Table S2.

Uncertain diplotype calls were evaluated on a case-by-case basis following predefined decision criteria. Visual inspection of genomic data was performed using the Integrative Genomics Viewer (IGV) software, based on Binary Alignment/Map (BAM) files and their corresponding index (BAI) files for each sequenced sample. Variant positions and star-allele definitions were obtained from the PharmVar database (https://www.pharmvar.org/). When diplotype ambiguity could not be resolved through read-level inspection or when no predefined star-allele definition matched the observed variant pattern, the sample was excluded from the analysis for that specific gene. The number of samples excluded due to unresolved or unmatched diplotype calls was 3 for *SLCO1B1*, 8 for *CYP2B6*, 1 for *CYP2C9*, 1 for *CYP3A5*, and 34 for *CYP4F2*.

The classification of actionable phenotypes of interest included poor function for *ABCG2*; decreased and poor functions for *SLCO1B1*; presence of pathogenic or likely pathogenic variants for *RYR1*; poor, intermediate, rapid and ultrarapid metabolizers for *CYP2B6* and *CYP2C19*; poor and intermediate metabolizers for *CYP2C9*, *CYP3A5*, *DPYD*, *NUDT15*, *TPMT* and *UGT1A1*; and poor metabolizers for *CYP3A4*.

### 2.4 Global ancestry inference

Global ancestry was inferred using the ADMIXTURE (Alexander et al., 2009) software by a supervised analysis for k = 4. To generate the reference panel, the multi-sample VCF file containing all target samples was merged with samples from the 1000 Genomes Project (Phase 3) for African, East Asian, and European populations and from the Human Genome Diversity Project (HGDP) for Native American continental populations (Bergström et al., 2020; Fairley et al., 2020). Hardy-Weinberg equilibrium (HWE)-normalized principal component analysis (PCA) (hwe_normalized_pca) was performed using Hail (hail.is) (Hail Team, 2025).

## 3 Results

**FIGURE 1:**
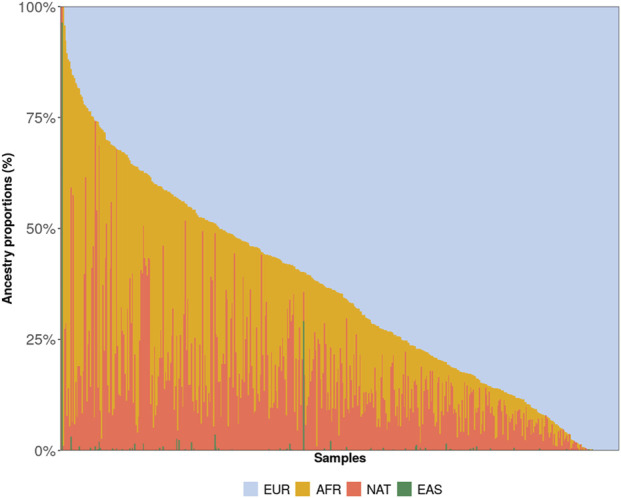
Genetic ancestry proportions estimated for all participants included in the study. NAT = Native American; AFR = African; EAS = East Asian; EUR = European.

Table 1 presents the demographic characteristics of the sample. Approximately half of the participants self-identified as White, and approximately 60% were born in the Southeast or South regions of Brazil. The median age at diagnosis was 64.47 (±9.60) years in men and 48 (IQR 41–57) years in women. Global ancestry inference revealed that the cohort was predominantly of European ancestry (64.32%, SD 23.63%), followed by African (20.60%, SD 18.79%) and Native American (14.69%, SD 12.58%) proportions, with East Asian ancestry representing <1% (Figure 1).

**TABLE 1:** Demographic characteristics of women with HER2+ BC and men with MPC.

Variable	Women with HER2+ BC (n = 259)	Men with MPC (n = 193)
Age (years), mean (SD)	52.2 (11.69)	68.4 (9.50)
Ethnicity*		
White	120 (49.38)	98 (50.78)
Black	32 (13.17)	22 (11.40)
Mixed	89 (36.63)	72 (37.31)
Asian	2 (0.82)	1 (0.52)
Brazilian macro-region of birth		
Central-west	16 (6.23)	11 (5.70)
Northeast	46 (17.90)	58 (30.05)
North	24 (9.34)	15 (7.77)
Southeast	106 (41.25)	52 (26.94)
South	65 (25.29)	57 (29.53)
Clinical comorbidities	120 (48.00)	118 (61.78)
Systemic arterial hypertension	66 (25.48)	97 (50.26)
Diabetes mellitus	32 (12.36)	33 (17.10)
Ischemic heart disease	3 (1.16)	11 (5.70)
Dyslipidemias	12 (4.63)	13 (6.74)
Hypothyroidism	22 (8.49)	5 (2.59)

Data are presented as n (%), unless otherwise stated. BC: breast cancer; MPC: metastatic prostate cancer. *Ethno-racial self-identification was obtained through interviews and classified according to the categories defined by the Brazilian Institute of Geography and Statistics (IBGE).

The prevalence of actionable PGx phenotypes in our sample was 99.33%. Only 1 woman with BC and 2 men with MPC exhibit no actionable phenotypes. Among participants with actionable phenotypes, the majority carried 2 (n = 124, 27.43%) or 3 (n = 144, 31.86%) phenotypes, with a maximum of 6 (n = 3, 0.66%). Allelic and genotypic prevalences, along with their respective phenotypes, are presented in Supplementary Tables S3, S4.

**FIGURE 2:**
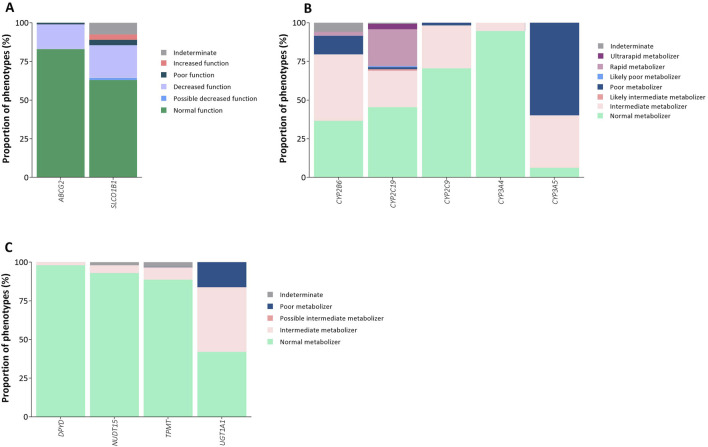
Actionable phenotype prevalence in Brazilian patients with PC and BC. **(A)** Drug transporter genes, **(B)** Cytochrome P450 (CYP) genes, **(C)** Other Drug-metabolizing enzyme genes. Stacked bar plots show the proportional distribution of phenotype categories for each gene, with percentages summing to 100% within each bar. Sample sizes were as follows: *ABCG2*, *DPYD*, *CYP2C19*, *NUDT15*, *TPMT*, *UGT1A1* (n = 452); *CYP3A5*, *CYP2C9* (n = 451); *SLCO1B1* (n = 450); *CYP3A4* (n = 446); *CYP2B6* (n = 444). Detailed counts of each phenotype category within each gene are provided in Supplementary Material.

A single variant was analyzed in 3 genes. The minor allele frequency of the *ABCG2*-rs2231142 T allele was 8.96%, which is associated with decreased function. Decreased and poor functions accounted for 16.15% (G/T genotype, n = 73) and 0.89% (T/T genotype, n = 4) of the predicted phenotypes, respectively (Figure 2A). The *IFNL3/4*-rs12979860 T-allele and the *VKORC1*-rs9923231 T-allele had frequencies of 40.82% and 32.63%, respectively.

A total of 17 *SLCO1B1* alleles were identified in this Brazilian sample (Supplementary Table S3), and normal function was the most frequent phenotype (n = 284, 63.11%). The prevalence of decreased function was 21.11% (n = 95), with a lower prevalence of increased function (n = 16, 3.56%) and poor function (n = 16, 3.56%) (Figure 2A; Supplementary Table S4). The most common star allele was the normal function SLCO1B1*37 (n = 179, 19.93%). Other observed alleles included the no function *15 (n = 116, 12.92%), and the increased function *14 (n = 93, 10.36%) and *20 (n = 58, 6.46%). Three missense variants in *SLCO1B1* are not currently included among PharmVar-reported alleles (Table 2). Each variant was rare, identified in a single individual, corresponding to a frequency of 0.11%.

**TABLE 2:** Missense variants identified in our sample that are not listed among PharmVar-reported alleles, along with their frequencies.

Gene	Protein change	Variant ID	Allele frequency	SIFT	PolyPhen
*SLCO1B1*	p.Ile237Val	rs1007241293	0.11%	Tolerated	Benign
​	p.Gly256Arg	rs754247932	0.11%	Deleterious	Probably damaging
​	p.Gln327His	rs151155254	0.11%	Tolerated	Benign
*CYP2B6*	p.Ser173Cys	rs148377536	0.33%	Tolerated	Benign
​	p.Ile209Val	rs144518874	0.33%	Tolerated	Benign
​	p.Glu339Ala	rs565104467	0.33%	Tolerated	Benign
​	p.His341Asp	rs138030127	0.33%	Tolerated	Benign
​	p.Val367Leu	rs143979776	0.33%	Tolerated	Benign
​	p.Arg323Gly	rs201282330	0.11%	Deleterious	Probably damaging
​	p.Thr67Met	rs138264188	0.44%	Deleterious	Probably damaging
​	p.Lys91Asn	rs772100005	0.22%	Tolerated	Benign
​	p.Asp257Asn	rs34646544	0.11%	Deleterious	Benign
​	p.Asp266Asn	rs770007043	0.11%	Deleterious	Benign
*CYP2C19*	p.Asn474Ser	rs1031294281	0.55%	Tolerated	Benign
​	p.Thr130Met	rs150152656	0.11%	Deleterious	Probably damaging
​	p.Val113Ile	rs145119820	0.22%	Tolerated	Benign
​	p.Ile60Thr	rs1848387737	0.11%	Tolerated	Benign
​	p.Glu81Lys	rs149072229	0.11%	Tolerated	Benign
​	p.Ile88Thr	rs1471083174	0.11%	Deleterious	Benign
​	p.Arg125His	rs141774245	0.11%	Tolerated	Benign
​	p.Arg132Trp	rs149590953	0.11%	Deleterious	Probably damaging
​	p.Ser336Ile	rs143833145	0.11%	Deleterious	Benign
​	p.Leu380Val	rs1364388853	0.11%	Tolerated	Benign
​	p.Asn423Lys	rs758626485	0.11%	Deleterious	Benign
*CYP2C9*	p.Glu104Asp	rs367848139	0.11%	Tolerated	Benign
​	p.Arg329Cys	rs768830601	0.11%	Tolerated	Possibly damaging
​	p.Ile389Thr	rs753971542	0.11%	Tolerated	Benign
​	p.His410Arg	rs746440122	0.11%	Deleterious	Benign
*CYP3A4*	p.Arg403Cys	rs143966082	0.22%	Deleterious	Probably damaging
​	p.Met395Val	rs142425279	0.11%	Tolerated	Benign
​	p.Thr310Lys	rs751246524	0.11%	Deleterious	Benign
*CYP3A5*	p.Asn247His	rs1810716398	0.11%	Deleterious	Benign
​	p.Ile149Thr	rs142823108	0.11%	Deleterious	Probably damaging
​	p.Ser100Tyr	rs41279857	0.22%	Deleterious	Probably damaging
*CYP4F2*	p.Ala483Gly	rs3952537	4.95%	Deleterious	Benign
​	p.Leu514Pro	rs777992718	0.11%	Deleterious	Probably damaging
​	p.Ser423Asn	rs780963140	0.22%	Tolerated	Benign
​	p.Ile406Thr	rs754740791	0.11%	Tolerated	Benign
​	p.His401Arg	rs1435072497	0.11%	Tolerated	Benign
​	p.Arg400Cys	rs75222722	0.22%	Deleterious	Probably damaging
​	p.Glu308Gly	rs200477560	0.11%	Deleterious	Probably damaging
​	p.Arg276Cys	rs61739998	0.11%	Tolerated	Benign
​	p.Asp265Asn	rs750598459	0.11%	Tolerated	Benign
​	p.Lys161Arg	rs138811366	0.33%	Deleterious	Possibly damaging
​	p.Ser2Phe	rs144146357	0.22%	Tolerated	Benign

Across *CYP* genes, intermediate metabolizer was the most prevalent phenotype in *CYP2B6* (n = 191, 43.02%), followed by the normal metabolizer (n = 162, 36.49%, Figure 2B). The reference *1 (n = 466, 52.48%) and the decreased function *6 (n = 254, 28.60%) alleles were the most common among the 19 star alleles identified in the sample. Five missense variants not included among PharmVar alleles were reported in the same three individuals with an allele frequency of 0.33%: rs148377536 (p.Ser173Cys), rs144518874 (p.Ile209Val), rs565104467 (p.Glu339Ala), rs138030127 (p.His341Asp), and rs143979776 (p.Val367Leu). Another unreported missense variant, rs201282330 (p.Arg323Gly), was found in one individual. Importantly, this individual was homozygous for the other two missense variants already reported among *CYP2B6* alleles (p.Lys262Arg and p. Gln172His). Another four missense variants not reported among PharmVar alleles were identified (Table 2).

Normal metabolizers were the most frequent phenotypes in *CYP2C19* (n = 205, 45.35%), *CYP2C9* (n = 318, 70.51%), and *CYP3A4* (n = 422, 94.62%) genes (Figure 2B). In the *CYP2C19* gene, similar prevalences of rapid (n = 108, 23.89%) and intermediate (n = 106, 23.45%) metabolizers were observed, and *1 (normal function, n = 573, 63.38%), *17 (increased function, n = 160, 17.70%) and *2 (no function, n = 115, 12.72%) alleles were the most prevalent. We identified eleven missense variants not included among *CYP2C19* PharmVar-reported alleles (Table 2). Interestingly, the unreported *CYP2C19* p. Thr130Met variant (rs150152656) and the *CYP2C19* p. Ile331Val variant (rs3758581) were in the same haplotype, since the individual was homozygous for *CYP2C19* p. Ile331Val. For the *CYP2C9* and *CYP3A4*, the *1 allele was the most common among the 10 and 8 different alleles identified, respectively (Supplementary Table S3). Among PharmVar unreported alleles, we found four missense variants in the *CYP2C9* gene, with an allele frequency of 0.11%, of which two were found in the same individual (*CYP2C9* p. Ile389Thr and p. His410Arg) (Table 2). For the *CYP3A4* gene, three missense variants (Table 2) and one frameshift variant (rs1263184572, p. Leu51PhefsTer39) were identified. The frameshift variant had an allele frequency of 0.11% and lacks *in silico* predictions in SIFT and PolyPhen databases.

On the other hand, poor (n = 270, 59.87%) and intermediate (n = 153, 33.92%) metabolizers were the most frequent phenotypes identified in the *CYP3A5* gene (Figure 2B; Supplementary Table S4). In this case, the nonfunctional CYP3A5*3 allele had a prevalence of 70.95% (n = 640). Among the alleles not reported in PharmVar, we identified three missense variants (Table 2) and two stop-gained variants in this gene. The *CYP3A5* p. Gln460* variant (rs149664815) was observed in one individual (0.11%), while the *CYP3A5* p. Arg268* variant (rs148176345) was detected in three individuals (0.33%).

Finally, in the *CYP4F2* gene, the *1/*1, *1/*4, and *1/*17 diplotypes were most prevalent, with no recommendations or predicted phenotypes determined by our analysis (Supplementary Table S3). Moreover, among missense variants in the *CYP4F2* gene, the *CYP4F2* p. Ala483Gly variant (rs3952537) showed an allele frequency of 4.95% (Table 2). Based on inferred phased genomic data, the *CYP4F2* p. Ala483Gly forms a haplotype with *CYP4F2* p. Thr472Ala, a variant of the reported CYP4F2*17 allele.

Across the analyzed pharmacogenes, most individuals were classified as normal metabolizers (Figure 2C). For *NUDT15*, 92.92% of participants (n = 420) showed a normal phenotype, predominantly the *1/*1 diplotype, while nonfunctional (*2, *3, *9) and uncertain function (*4, *6) alleles were rare. Similarly, for *TPMT*, the *1/*1 diplotype was the most frequent (n = 401, 88.72%), although 37 individuals carried nonfunctional alleles (*2, *3A, *3C, *8, *9, *24, *34) and were classified as intermediate, possible intermediate, or poor metabolizers. For *UGT1A1*, the distribution was more balanced, with comparable frequencies of normal (42.0%) and intermediate (41.8%) metabolizers, while decreased-function alleles (*6, *28, *80+*28, *80 + 37) accounted for approximately 37% of the cohort.

In *DPYD*, intermediate metabolizers were uncommon (n = 9, 2.0%). The nonfunctional DPYD*2A, decreased function rs67376798-A, and HapB3 variants were each detected in 0.33% of participants, while *7 and rs115232898 were each in 0.11%. In *RYR1*, malignant hyperthermia susceptibility was identified in only 2 individuals (0.44%), while 98.76% (n = 446) exhibited normal function. Variants of uncertain function (c.652G>A; c.9635A>G; c.12553G>A; c.1598G>A) were identified in 4 participants (0.88%). Moreover, 2 undocumented genetic variations were detected in our sample. Forty-five individuals had a T-allele at the *RYR1* c.7089 position, and 1 individual had a T-allele at the *RYR1* c.6612 position (rs141646642). Importantly, the *RYR1* c.7089C>G and c.6612C>G variants have been described as associated with malignant hyperthermia susceptibility. Detailed allele frequencies are provided in Supplementary Tables S3, S4.

## 4 Discussion

In this study, we conducted a comprehensive PGx analysis in a cohort of Brazilian patients with MPC and HER2-positive BC using exome sequencing and the PharmCAT. Nearly all participants carried at least one actionable PGx phenotype, underscoring the potential clinical utility of PGx testing in this population. In settings characterized by polypharmacy, narrow therapeutic indices, and cumulative toxicities, preemptive genotyping offers a realistic path to fewer adverse events, more predictable exposures, and more efficient use of supportive care. Evidence from population-scale studies has shown similar clinical relevance across diverse cohorts (Bousman et al., 2025; Hodel et al., 2024; McInnes et al., 2021; Moore et al., 2025), reinforcing the generalizability of our conclusions to routine cancer care. The high prevalence of actionable variants across multiple genes highlights the importance of incorporating PGx testing into treatment decision-making, particularly in oncology, where polypharmacy and drug toxicity are common. Our results revealed substantial variability in the frequency of actionable alleles across pharmacogenes, consistent with findings from a previous study in a Southeastern Brazilian cohort (Bertholim-Nasciben et al., 2023).

*ABCG2*, encodes the BC resistance protein (BCRP), a major efflux transporter involved in drug predisposition. The common missense variant rs2231142 (Q141K) leads to the substitution of glutamine (Q) by lysine (K) at position 141, resulting in decreased transport activity (Suominen et al., 2023). In our cohort, 17% of individuals carried the T allele associated with decreased function, which may increase exposure to multiple oncology and supportive-care drugs. The clinical consequences are setting- and drug-dependent, and may include high rates of cytopenias, diarrhea, and elevated liver function tests in susceptible patients, in addition to an increased risk of rosuvastatin-induced myopathy, particularly in the presence of other risk factors or drug–drug interactions (Suominen et al., 2023).

The *IFNL3/4* rs12979860 T allele is a well-established predictor of response to interferon-based therapy in chronic hepatitis C virus (HCV) infection. Its clinical relevance in oncology, however, remains limited and has not been definitively established. Most evidence derives from studies in patients with chronic HCV infection, where the T allele has been associated with a higher risk of hepatocellular carcinoma and with differences in immune–inflammatory pathways that may influence cancer susceptibility or progression. Beyond this context, its impact on cancer treatment outcomes or toxicity remains exploratory, with no actionable oncology-specific guidelines currently available. Therefore, to date, there is no evidence supporting its role in systemic therapy selection for BC or prostate cancer (PC), and it should not guide oncology decisions (Hou et al., 2019). Conversely, the *VKORC1* rs9923231 T-allele, identified in one-third of our cohort, is associated with increased warfarin sensitivity, conferring lower dose requirements. This finding may impact patients with cancer, who often require warfarin for venous thromboembolism prophylaxis or treatment (Johnson et al., 2017).

*SLCO1B1* encodes the hepatic uptake transporter Organic Anion Transporting Polypeptide 1B1 (OATP1B1), which mediates the hepatic uptake of several statins. Decreased/poor function (often driven by *5/*15 haplotypes) increases systemic statin exposure and the risk of myopathy, especially with simvastatin and, to a lesser extent, atorvastatin (Ramsey et al., 2014). In our sample, *SLCO1B1* exhibited marked allelic heterogeneity, with decreased-function variants accounting for 21.1% of predicted phenotypes, consistent with frequencies reported in Latin American populations (Cooper-DeHoff et al., 2022; Ramsey et al., 2014). OATP1B1 also contributes to the hepatic uptake of docetaxel, and some studies have related *SLCO1B1* variants/haplotypes to altered docetaxel pharmacokinetics and toxicity. However, these findings are inconsistent and currently do not support genotype-based dosing (Hjorth et al., 2022; Schmidt et al., 2025).

*CYP* genes accounted for most of the predicted metabolic variability. For instance, CYP2B6 contributes to the activation and clearance of several drugs commonly used in oncology, such as cyclophosphamide in combination regimens, bupropion, and methadone (Zanger and Klein, 2013). In patients with intermediate or reduced CYP2B6 activity receiving cyclophosphamide, which is frequently used in the neo/adjuvant treatment of BC, closer monitoring for myelosuppression and nausea/vomiting is recommended, along with the evaluation of concomitant medications that may inhibit or induce CYP2B6 activity (Helsby et al., 2021). In our cohort, *CYP2B6* exhibited substantial allelic heterogeneity, with 19 different star alleles identified. The prevalence of the *6 allele is consistent with frequencies reported in European and Latin American populations, ranging from 15% to 30% (Langmia et al., 2021; Zanger and Klein, 2013; Zhou and Lauschke, 2022).

The distribution of *CYP2C19* alleles in our sample and the resulting phenotype diversity paralleled findings from admixed and European cohorts, reinforcing the genetic heterogeneity of the Brazilian population (Zhou and Lauschke, 2022). *CYP2C19* loss-of-function alleles (e.g., *2) reduce clopidogrel activation and increase the risk of stent thrombosis; whereas the *17 allele is associated with increased function, which may increase bleeding risk and reduce proton-pump inhibitor exposure, potentially compromising reflux prophylaxis or ulcer prevention. CYP2C19 activity also influences the metabolism of several antidepressants prescribed for hot flashes, anxiety, or depression (e.g., citalopram/escitalopram, sertraline) (Scott et al., 2013). In the context of MPC treatment, enzalutamide acts as a moderate CYP2C19 inducer, which may further reduce exposure to proton-pump inhibitors and antidepressants in *17 carriers who are already predisposed to faster CYP2C19 metabolism (Gibbons et al., 2015).

*CYP3A5* showed marked variability, which aligns with previous findings that *CYP3A5* loss-of-function alleles are common in European-derived populations, but less frequent in African populations, where functional alleles are more common (PharmGKB, 2025). CYP3A5*6 and *7 alleles were slightly more prevalent in our cohort that in European ancestry populations, aligning with previous estimates from Latino populations (Pratt et al., 2023). Although CYP3A4 is the dominant enzyme in the taxane pathway, *CYP3A5* loss-of-function alleles may reduce overall CYP3A capacity and exacerbate toxicity when CYP3A4 activity is concurrently inhibited. Moreover, studies have supported an association of PC risk with *CYP3A5* variants, as well as with increased *CYP3A5* expression (Van Eijk et al., 2019).

For *CYP2C9*, the prevalence of decreased-function alleles translated into 29.5% of individuals being predicted as poor or intermediate metabolizers, in line with global estimates (Zhou et al., 2023). Although less common in our cohort, *CYP2C9* loss-of-function alleles (*3, *6, *33) are known to reduce warfarin clearance, thereby increasing bleeding risk at standard doses (Sanderson et al., 2005).

In genes such as *TPMT*, *NUDT15*, and *DPYD*, although the prevalence of nonfunctional alleles was low, the clinical implications remain substantial, as carriers are at a significantly increased risk of severe toxicity when exposed to thiopurines or fluoropyrimidines. Particularly, the *NUDT15* nonfunctional alleles *2, *3, and *9 —associated with an intermediate metabolizer phenotype—were rare, and the data are consistent with findings in European, Amazonian Amerindian, and admixed Brazilian cohorts (Karczewski et al., 2020; Naslavsky et al., 2022; Rodrigues et al., 2020). Similarly, TPMT and DPYD poor and intermediate metabolizers were less frequent, confirming previous findings in Brazilian cohorts (Bertholim-Nasciben et al., 2023; Naslavsky et al., 2022). There is robust evidence that genotype-guided fluoropyrimidine dosing significantly reduces grade ≥3 toxicity (Amstutz et al., 2018) and converging regulatory frameworks support pre-treatment *DPYD* testing (Keen et al., 2025). Routine implementation of this practice is warranted in Brazilian BC care settings, with dose reductions according to international guidelines (Amstutz et al., 2018). Finally, the *UGT1A1* gene showed decreased-function alleles in approximately 37% of participants, resulting in a large proportion of predicted intermediate metabolizers, consistent with a previous study in a Brazilian population (Bertholim-Nasciben et al., 2023). This may be clinically relevant when using drugs whose exposure/toxicity is driven by UGT1A1 activity, such as sacituzumab govitecan and irinotecan (Karas and Innocenti, 2022).

The high prevalence of actionable PGx profiles in our sample (99.3%) has significant implications for precision oncology within the Brazilian public health system (SUS). Patients with BC and PC are frequently exposed to drugs with narrow therapeutic indices and high risk of adverse reactions, including tamoxifen, fluoropyrimidines, taxanes, statins, and anticoagulants (Al-Mahayri et al., 2020; Basak et al., 2021). In this context, *DPYD*, *NUDT15* and *TPMT* variants may predict chemotherapy-related toxicity (Larrue et al., 2024; Yang et al., 2015). Since 2020, the European Medicines Agency has recommended dihydropyrimidine dehydrogenase (DPD) deficiency testing before the use of fluorouracil and capecitabine (De With et al., 2023), and the U.S. Food and Drug Administration has recently updated the product labeling of these drugs, to increased awareness of DPD deficiency (FDA, 2025). Additionally, testing for *TPMT* and *NUDT15* genes has been funded by the United Kingdom National Health Service for patients with acute lymphoblastic leukemia treated with purine analogs (NHS, 2025). In Brazil, there is no formal recommendation or mandatory coverage for *TPMT*, *NUDT15,* or *DPYD* testing by national health authorities.

The relevance of our results lies in the admixed genetic background of the Brazilian population, characterized by different contributions from European, African, and Native American ancestries. Our ancestry analysis confirmed this admixture and identified rare and previously unreported variants, further supporting the notion that admixed populations remain underrepresented in global PGx databases (Mychaleckyj et al., 2017; Secolin et al., 2019). This underrepresentation may limit the accuracy of phenotype prediction tools and clinical guidelines, which have been developed using predominantly European reference populations. Our study, therefore, contributes to filling this knowledge gap by providing large-scale data from a representative Brazilian cohort. From a clinical perspective, several of the PGx variants identified in this study are included in existing pharmacogenomic guidelines and may help inform treatment decisions. Although formal clinical impact was not evaluated here, these findings highlight the potential relevance of integrating PGx information into public healthcare settings.

Strengths of our study include the use of exome sequencing, which enabled the detection of both common and rare alleles, the multicenter recruitment strategy ensuring representation from all Brazilian macro-regions, and the application of standardized tools (PharmCAT and CPIC® guidelines) for variant interpretation. Nevertheless, some limitations must be acknowledged. First, *CYP2D6*, a clinically relevant pharmacogene, was not assessed due to technical challenges in accurately detecting copy number and structural variants using exome sequencing. More broadly, exome sequencing primarily targets coding regions and does not reliably capture promoter, intronic, or other structural variants, which may also be functionally relevant in pharmacogenomics. As a result, regulatory and structural variation contributing to drug response may not be fully represented in this study. Second, although novel missense variants were identified, their functional impact could not be reliably inferred based solely on sequence data. Functional validation and predictive modeling will be required to determine their potential clinical relevance. Third, the study population consisted exclusively of patients with cancer, which may not fully represent the general Brazilian population. Finally, the predominance of European ancestry in our cohort, while reflective of certain regions of Brazil, may not capture the full extent of variability across the country.

In conclusion, our study highlights PGx variability in a large admixed Brazilian cohort, consistent with global data but shaped by the unique ancestry composition of the population. Our data also demonstrate that nearly all individuals harbor actionable PGx phenotypes. Incorporating PGx testing into SUS protocols could improve patient safety, reduce adverse drug reactions and hospitalizations, and optimize therapeutic outcomes. However, such implementation requires overcoming barriers such as financial constraints, infrastructure, and training healthcare professionals. These results also emphasize the urgent need to continue generating genomic data from underrepresented populations. Such efforts will be essential for the equitable advancement of precision medicine worldwide.

## Data Availability

The datasets presented in this article are not readily available because This data is part of the Programa Genomas Brasil of the Brazilian Ministry of Health. Allele frequencies for the studied genes are available in the Supplementary Material. Further data can be obtained by contacting the authors. Requests to access the datasets should be directed to GM, gabriel.macedo@hmv.org.br.
